# Gastric Stem Cells: Physiological and Pathological Perspectives

**DOI:** 10.3389/fcell.2020.571536

**Published:** 2020-09-17

**Authors:** Shiyu Xiao, Liya Zhou

**Affiliations:** ^1^Department of Gastroenterology, Peking University Third Hospital, Beijing, China; ^2^Beijing Key Laboratory of Helicobacter Pylori Infection and Upper Gastrointestinal Diseases, Peking University Third Hospital, Beijing, China

**Keywords:** gastric stem cells, gastric mucosa homeostasis, stem cell niche, gastric organoid, Helicobacter pylori, stomach neoplasms

## Abstract

Gastric epithelium operates in a hazardous environment that curtails the lifespan of the constituent cells, imposing a requirement for continuous epithelial renewal. Stem cells that reside in the stomach are thus essential for regulating physiological tissue renewal and injury repair because of their self-renewal, high proliferation capacity and multiple differentiation potentials. Recent investigations using lineage tracing models have identified diverse populations of gastric stem cells and even fully differentiated cells that can regain stem cell capacity, so enriching our understanding on the identity and plasticity of gastric stem cells. These cell populations include the Villin promotor, Lgr5^+^, CCKR2^+^, Axin2^+^ and AQP5^+^ stem cells in the antrum, TFF2 mRNA, Mist1^+^ cells and Troy^+^ mature chief cells in the corpus, as well as Sox2, eR1, Lrig1, Bmi1-marked cell in both the antrum and the corpus. Establishment of gastric organoids derived from primary gastric tissues and pluripotent stem cells or embryonic stem cells characterizes niche factors required by the gastric stem cell populations, and further provides new insights into stomach development, host-Helicobacter pylori interactions and malignant transformation. Furthermore, focus on the gastric stem cells and their niches uncovers the initiation of stomach precancerous lesions and origin of gastric cancer, providing options for cancer prevention and intervention. In summary, with the development of stem cell research, gastric stem cells give us more opportunities to prevent and treat stomach diseases.

## Introduction

The stomach epithelium is a hazardous environment that curtails the lifespan of constituent cells, imposing a requirement for continuous renewal of epithelium. Stems cells in our gut are, thus, essential for epithelial regeneration and damage repair owing to their ability for self-renewal, high proliferation capacity and potential for multiple differentiation. Gastric stem cells, a group of adult stem cells residing in the stomach, play a key role in maintaining the dynamic homeostasis of the gastric epithelium (Mills and Shivdasani, 2011). In recent years, advances have been made in the investigation of molecular markers identifying gastric stem cells. In addition, *in vitro* gastric stem cell models have been established, revealing the role of these cells in physiology and pathology. Although some sporadic reviews on this topic have been published in past years (Bartfeld and Koo, 2017; Hata et al., 2018), this present review aim to provide fresh and profound insights into stomach stem cells from physiological and pathological perspectives.

## Properties of Gastric Stem Cells

Stems cells are a group of cells defined by their ability of self- renewal and multi-potency, which can be divided into embryonic stem cells and adult stem cells in terms of their development stage. Tissue-resident adult stem cells are a small population of adult stem cells, these specialized cells are particular important in the epithelium lining of the alimentary tracts and skin that require constant dynamic replacement of the epithelial population (Barker et al., 2010a). More importantly, given their ability of directional differentiation, tissue-resident stem cells are responsible for tissue homeostasis, injury repair, and even cancer development.

Gastric stem cells represent an adult stem cell population residing in the stomach tissues with high proliferative potential, which enables efficient stomach epithelium regeneration and repair. Following the comprehensive investigation of intestinal stems cells, the identity of gastric stem cells is being explored. In comparison with intestinal stems cells, gastric stem cells share many properties, but they differ in fundamental aspects regarding location, molecular cell markers and their specific growth niches.

## Identity of Gastric Stem Cells

### Location of Gastric Stem Cells

**FIGURE 1:**
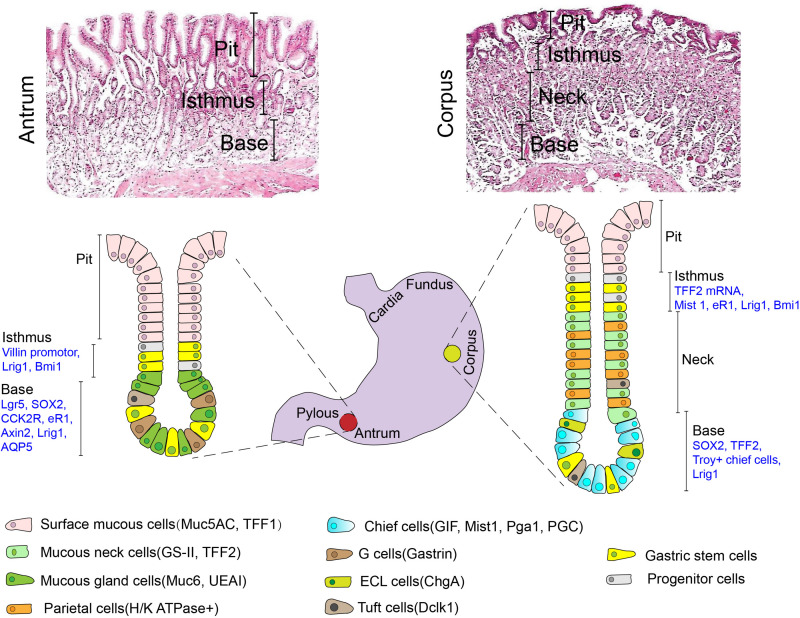
Typical H&E histology and a schematic depicting the structure and cell types (including mature cells with their specific molecular markers and candidate stem cells) of gastric glands in different anatomical regions (Antrum vs. Corpus). Muc5AC mucin 5AC, TFF1 trefoil factor 1, GS-II griffonia simplicifolia II, TFF2 trefoil factor 2, Muc6 mucin 6, UEAI ulex europaeus agglutinin I, GIF gastric intrinsic factor, Pga1 pepsinogen 1, PGC pepsinogen C, ChgA chromogranin A, Dclk1 double-cortin-like kinase 1.

The mucosa in all parts of the human stomach is lined by a simple columnar epithelium that has numerous tubular invaginations in its lamina propria. These invaginations, termed gastric units, consists of a pit, isthmus, neck and the base regardless of different anatomical zone, although their cellular composition varies with the region of the stomach in which they are located (Lee et al., 1982; Choi et al., 2014). Five types of differentiated mature cells, namely surface mucus cells, mucus neck cells, parietal cells, chief cells, enteroendocrine cells (including G cells, D cells, and ECL cells) and tuft cells, make up gastric glands. However, the mesenchymal compartment surrounding the glands is less studied and little understood. A schematic diagram depicting the structure and cell type of gastric glands in different regions is presented in Figure 1. Under physiological conditions, gastric epithelial cells undergo continuous dynamic renewal within as little as 3 days (Karam and Leblond, 1993b). Consequently, gastric epithelial stem cells are essential for the regeneration of lost or damaged cells in stomach mucosa. An understanding of the location of adult stem cells in the stomach is, therefore, important to explore their function.

Previous studies, using nucleotide incorporation assays and ultrastructural analysis, have demonstrated that the isthmus might be the pool of stem-like cells in an adult stomach (Leblond et al., 1948; Corpron, 1966; Karam and Leblond, 1993a). This group of cells produces descendants that undergo a complex bi-directional migration toward pit and base. However, direct evidence, describing their route of differentiation and migration, remains elusive. Then, Bjerknes and Cheng (2002), for the first time, used transgenic mice expressing a bacterial gene for β-galactosidase (lacZ) under a Rosa26 promotor (for visualization) and random chemical mutagenesis to demonstrate the existence of long-lived committed progenitors or stem cells in gastric epithelium. Cells at the base of the gastric glands were further identified as a second stem cell pool and were capable of self-renewing and differentiating. In addition, the emergence of lineage tracing models has made it possible for us to define the direction of differentiation of stomach stem cells in specific anatomic regions under normal or abnormal conditions.

### Gastric Stem Cell Marker Candidates

In early studies, the identity of potential stem cells in the stomach was mainly based on their presumed morphological characteristics and cell proliferation kinetics. With the application of lineage tracing approaches combined with *in situ* hybridization or immunohistostaining, an array of biomarkers is being proposed for characterizing the population of adult stem cells in different anatomic regions of the stomach (Figure 1).

In the antral and pyloric glands, several markers are identified to be specific to this zone. Villin promotor is an epithelial cell-specific, calcium-regulated actin-binding protein that modulates reorganization of microvillar actin filaments. Qiao et al. showed that Villin promotor-marked cells represent a highly quiescent stem cell that only becomes apparent upon stimulation by interferon γ (Qiao et al., 2007). This group of stem cells is mainly located in the third part of the glands, with lineage tracing showing that they can differentiate into all types of cells in the antral glands. Lgr5 (leucine-rich repeat-containing G-protein coupled receptor 5) is a widely-accepted marker of stem cells in the gastrointestinal tract (Koo and Clevers, 2014). Baker et al. demonstrated that Lgr5^+^ cells at the very base of pyloric glands were long-term, self-renewing and multipotent stem cells, responsible for maintaining gastric epithelial homeostasis (Barker et al., 2010b). They could differentiate into surface mucus cells, enteroendocrine cells and parietal cells. Lgr5 expression was also found in a subpopulation of chief cells at the antral base, where these Lgr5^+^ chief cells functioned as damage-induced stem cells for gland regeneration following injury (Leushacke et al., 2017). In this same zone, a group of cells is Axin2^+^/Lgr5^–^ stem cells that can give rise to Lgr5^+^ cells (Sigal et al., 2017). They are characterized by a high proliferation rate in the dependence of Wnt agonist-R spondin. Cholecystokinin type-B receptor (CCK2R)-positive cells are another set of stem cells which does not overlap with Lgr5^+^ cell population at the antral gastric units, and treatment with progastrin interconverts CCKR2^+^ Lgr5^neg or low^ antral stem cells into typical antral Lgr5^high^ cells that expand the active stem cells in the antrum (Hayakawa et al., 2015b). More recently, AQP5 expressing populations are identified to overlap with Lgr5^+^ stem cells at the base of pyloric gland, where this population of stems cells comprises the pyloric lineages expressing Muc5AC, gastric intrinsic factor, gastrin and chromogranin A (Tan et al., 2020).

In contrast to the antral glands, TFF2 (trefoil factor family 2) transcript, Mist1 and Troy (tumor necrosis factor receptor superfamily, member 19) may be as the candidate of the adult stem cell markers in the oxygenic mucosa. Lineage tracing demonstrated that TFF2 mRNA-expressing cells above the neck region are the progenitors for mucus neck, parietal and chief cells but not for pit or ECL cell lineages in the corpus (Quante et al., 2010), while TFF2 protein is mainly expressed and secreted by mucus neck cells and deep antral gland mucus cells under normal condition (Farrell et al., 2002). Within the corpus isthmus, Mist1^+^/Lgr5^–^ cells are also capable of giving rise to surface mucus cells, mucus neck cells, parietal cells, tuft cells and ECL cells (Hayakawa et al., 2015a; Nienhüser et al., 2020). *In vitro*, Mist1^+^ isthmus cells can form corpus organoids, but Mist1^+^ chief cells remain single (Hayakawa et al., 2015a). This evidence suggests that isthmus Mist1^+^ cells play a critical role in differentiation of corpus glands. In contrast to those with the properties of stemness and self-renewal, a subgroup of fully differentiated cells has been found capable of regaining stem cell capacity. Troy^+^ differentiated chief cells at the base of gastric corpus units are considered reserve quiescent stem cells that display plasticity in that they are capable of re-entering the cell cycle to give rise to all gastric units (Stange et al., 2013). This result provides the evidence that differentiated cells in the stomach also participate in maintaining epithelial renewal and homeostasis through dedifferentiation or transdifferentiation.

Some biomarkers are shared among gastric stem cells in both pyloric and corpus glands. Sox2 (Sex-determining region Y box protein 2)-positive cells are found in both the pylorus and corpus of the glandular stomach in mice, although no apparent overlap exists with Lgr5^+^ cells in the pylorus (Arnold et al., 2011). Cells labeled by eR1 (a Runx1 enhancer element) in the isthmus of corpus and the base of pyloric gland are reported to be involved in tissue regeneration and continuously give rise to mature cells that maintain gastric units (Matsuo et al., 2017). Lrig1 (Leucine-rich repeats and immunoglobulin-like domain 1)-marked cells are known to give rise to daughter cells, and Lrig1-expressing isthmal cells can contribute to the regeneration of parietal cells following acute gastric injury (Choi et al., 2018). Bmi1-expressing cells in the isthmus of gastric antrum and corpus also provide progeny bipolarly toward luminal and basal sides, although it is not clear whether they are colocalized with other reported stem cells (Yoshioka et al., 2019). These candidate markers mentioned above and their relevant studies are concisely summarized in Table 1.

**TABLE 1:** Gastric stems cells labeled by candidate markers.

Markers	Location pattern	Differentiation by lineage tracing	Physiological characteristics	Organoid formation
Villin promotor (Qiao et al., 2007)	At or below the isthmus in the bottom third of pyloric glands	Mucus neck cells, mucus gland cells, parietal cells, enteroendocrine cells	Do not contribute to epithelial renewal under normal homeostatic conditions; respond to interferon γ	No
Lgr5 (Barker et al., 2010b; Stange et al., 2013; Leushacke et al., 2017)	Base of pyloric glands, a subpopulation of chief cells	Surface mucous cells, parietal cells, enteroendocrine cells	Maintain epithelial renewal in pyloric regions under normal homeostatic conditions; Lgr5^+^ adult stem cells originate from fetal Lgr5^+^ progenitors; neonate Lgr5^+^ cells contribute to the development of mature gastric epithelium in both pylorus and corpus regions; respond to Wnt agonist R-spondin; Be activated by acetylcholine-producing tuft cells through muscarinic receptor subtype 3	Yes
TFF2 mRNA (Quante et al., 2010)	Isthmus of corpus gland	Mucous neck cells, chief cells, parietal cells	Amplified by DMP-777 (chemicals inducing acute parietal cells loss); not the cell of origin for SPEM	No
Sox2 (Arnold et al., 2011)	Base of pyloric and corpus gland	Surface mucous cells, chief cells, parietal cells, enteroendocrine cells	Half of the Sox2^+^ cells is quiescent under homeostatic conditions; early Sox2^+^ fetal progenitor are the precursors for Sox2^+^ adult stem cells; no overlap with Lgr5^+^ cells in the pylorus	No
Troy (Stange et al., 2013)	Differentiated chief cells at the base of corpus gland	Surface mucous cells, mucous neck cells, chief cells, parietal cells, enteroendocrine cells	A reserve stem cell population; Wnt-driven stem cells	Yes
Mist1 (Hayakawa et al., 2015a; Nienhüser et al., 2020)	Isthmus of corpus gland	Surface mucous cells, mucous neck cells, parietal cells, enteroendocrine cells, tuft cells	Most Mist1^+^ stem cells in isthmus are quiescent under normal condition; kras mutation promotes Mist1^+^ stem cells proliferation and division; give rise to the entire gland in the response to gastric injury caused by acute damage and chronic inflammation	Yes
CCK2R (Hayakawa et al., 2015b)	Above the Lgr5^+^ cells (+ 4) at the base of antral gland	Surface mucus cells, G cells, D cells, tuft cells	No overlap with Lgr5^+^ stem cells; more rapid cycling than Lgr5^+^ cells; CCK2R^+^Lgr5^neg or low^ cells can convert to Lgr5^high^ cells following progastrin treatment; gastrin secreted from antral G cells regulates CCK2R^+^ stem cell function in a paracrine manner	Yes
eR1 (Matsuo et al., 2017)	Isthmus in the corpus gland, base of antral gland	Surface mucous cells, mucous neck cells, chief cells, parietal cells	Maintain the integrity of gastric units; play a role in tissue regeneration following tamoxifen treatment	Yes
Axin2 (Sigal et al., 2017)	Base and lower isthmus of antral gland	Surface mucous cells, mucous neck cells, enteroendocrine cells, tuft cells	Overlap with Lgr5^+^ stem cells; repopulation time from Axin2^+^ cells are rapid than Lgr5 + cells; Axin2^+^Lgr5^–^ cells can give rise to Lgr5^+^ cells; high proliferation rate of Axin2^+^ cells depend on Wnt agonist R spondin	Yes
Lrig1 (Choi et al., 2018)	Isthmus and base in the corpus and antral gland	Surface mucous cells, mucous neck cells, chief cells, parietal cells, G cells, tuft cells	Early Lrig1^+^ fetal progenitor are the precursors for Lrig1^+^ adult stem cells; reconstitutes gastric epithelium after acute oxyntic atrophy Do not give rise to metaplasia lineage	No
Bmi1 (Yoshioka et al., 2019)	Isthmus of pyloric and corpus gland	Surface mucous cells, mucous neck cells, chief cells, enteroendocrine cells, tuft cells	No overlap with Lgr5^+^ or eR1^+^ cells; provide progeny bidirectionally toward both the luminal and basal sides in the antrum and corpus; be required for the homeostasis and regeneration of gastric epithelium	Yes
AQP5 (Tan et al., 2020)	Base of pyloric gland	Surface mucous cells, parietal cells, enteroendocrine cells	Overlap with Lgr5^+^ populations	Yes

*Lgr5 leucine-rich repeat-containing G-protein coupled receptor 5, TFF2 trefoil factor 2, Sox2 sex-determining region Y box protein 2, CCK2R cholecystokinin type-B receptor, Lrig1 leucine-rich repeats and immunoglobulin-like domains 1, AQP5 aquaporin 5.*

Current available studies reveal a varying pattern in locations of gastric stem cells across different anatomical regions, indicating the complicated nature of gastric gland organization (Table 2). A recent study using lineage-tracing assays confirmed that gastric corpus gland is compartmentalized, with isthmus and base zones supported by two separate independent groups of stem cell, suggesting their specific molecular identity and functional behavior (Han et al., 2019). Future studies will be warranted to define the true identity and origin of stem cells in different regions, and unveil their specific roles in gastric mucosa homeostasis.

**TABLE 2:** Comparison of gastric stem cells in the antrum and corpus.

	Pyloric gland	Corpus gland
Cell location	Isthmus, Gland base	Isthmus, Gland base, certain differentiated cells (chief cells)
Morphology	No evidence	No evidence
Direction of differentiation	Stem cells derived clones expand bidirectionally for isthmus stem cells. For stem cells at the gland base, they migrating upward from the basal zone	Stem cells derived clones expand bidirectionally for isthmus stem cells. For stem cells at the gland base, they migrating upward from the basal zone
Molecular markers	Isthmus: Villin promotor, Lrig1, Bmi1 Gland base: Lgr5, SOX2, CCK2R, eR1, Axin2, AQP5	Isthmus: TFF2 mRNA, Mist1, eR1, Lrig1, Bmi1 Gland base: SOX2, TFF2, Lrig1, Troy^+^ chief cells
Offspring	Surface mucous cells, Mucous gland cells, G cells, Tuft cells	Surface mucous cells, Mucous neck cells, parietal cells, ECL cells
Known niche factors	Wnt, Notch, Gastrin, Ach, EGF, FGF10	Wnt, BMPs, Shh, innate lymphoid cells, endothelial cells, EGF, FGF10

*Lgr5 leucine-rich repeat-containing G-protein coupled receptor 5, TFF2 trefoil factor 2, Sox2 sex-determining region Y box protein 2, CCK2R cholecystokinin type-B receptor, Lrig1 leucine-rich repeats and immunoglobulin-like domains 1, AQP5 aquaporin 5, Ach acetylcholine, EGF epidermal growth factor, FGF10 fibroblast growth factor 10, BMPs bone morphogenetic proteins, Shh sonic hedgehog.*

### Gastric Stem Cells and Their Niche

A specific microenvironment is the key determinant to regulate biological behavior of stem cells. The niches consist of stem, stroma, and immune cells, as well as various growth signals, and extracellular matrices. Interactions between these components, therefore, contribute to proliferation of stem cells and directional differentiation. In the setting of gastric stem cell niche (Figure 3), little is known because of the complexity of gastric glands in different regions.

**FIGURE 2:**
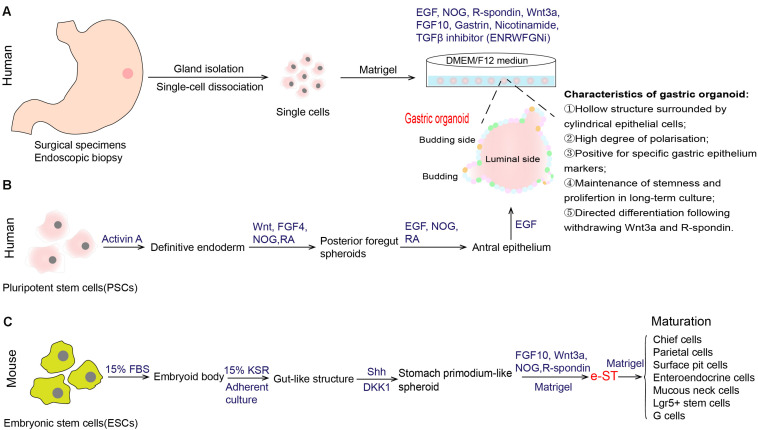
Establishment of *in vitro* culture system of gastric stem cells. **(A)** Generation of human gastric organoid from gastric glands that are derived from surgical specimens or endoscopic biopsy (Bartfeld et al., 2015). This organoid model could achieve long-term *in vitro* culture. **(B)** Generation of gastric organoid from human pluripotent stem cells (PSCs) (McCracken et al., 2014; Broda et al., 2019). **(C)** Generation of stomach primordium-like spheroids (named e-ST) from mouse embryonic stem cells (ESCs) (Noguchi et al., 2015). e-ST could differentiate into mature stomach tissue cells in Matrigel-based culture supplemented with FGF10, Wnt3a, NOG, and R-spondin. EGF epidermal growth factor, NOG Noggin, FGF 10 fibroblast growth factor 10, TGF-β transforming growth factor beta, FGF 4 fibroblast growth factor 4, RA retinoic acid, FBS fetal bovine serum, KSR knockout serum replacement, Shh sonic hedgehog.

**FIGURE 3:**
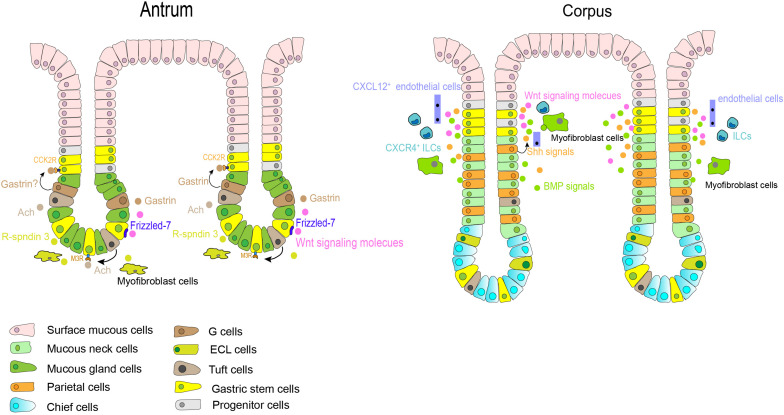
Gastric stem cells in different anatomic sites and their niche factors. In the antrum, Notch signals affect the balance between stem cell proliferation and differentiation. Gastrin and acetylcholine (Ach) also contribute to niche factors in this region. In corpus, Shh and BMPs signals are restricted to the isthmus and neck of fundic gland. Stromal cells, such as Cxcl12 + endothelial cells and Cxcr4 + innate lymphoid cells, also contributes to the corpus stem cell niche partly through production of Wnt5a. Other growth factors, such as Wnt, EGF, and FGF10, play an important role in regulation of self-renewal and differentiation of gastric stem cells in the base gland of antrum and the isthmus of corpus.

Some of the major regulators of gastric stem cell proliferation and differentiation include sonic hedgehog (Shh) and bone morphogenetic proteins (BMPs) signaling pathways. These have been found to exert important effects on gastrointestinal development and differentiation (Lees et al., 2005; Todisco, 2017). In the human stomach, Shh expression is restricted to the parietal cells and the glandular portion (van den Brink et al., 2001), where it controls epithelial cell proliferation and serves as a polarizing signal for fundic gland differentiation partially through autocrine (van den Brink et al., 2001, 2002). BMPs are regulatory peptides that are mainly secreted from interstitial myofibroblast-like cells (van den Brink et al., 2001; Takabayashi et al., 2014), the isthmus and neck of the glands are the targeted location receiving BMPs-generated signals (Takabayashi et al., 2014). A series studies have indicated that dysregulation of BMP signaling in the stomach can lead to perturbations of normal homeostatic mechanisms of the gastric mucosa, resulting in developing metaplasia, dysplasia and even neoplasia (Todisco, 2017). Notch signaling is another important signal in this microenvironment (Kim and Shivdasani, 2011). Activation of this pathway directly regulates proliferation of Lgr5^+^ antral stem cells via NOTCH1 and NOTCH2 receptors, but results in a decrease in antral cellular differentiation (Demitrack et al., 2017; Gifford et al., 2017). In the gastric antrum, therefore, Notch might affect the balance between stem cell proliferation and differentiation and maintain homeostasis of gastric epithelium. Thus, abnormal activation of Notch signals can promote the transformation of gastric stem cells to cancer stem cells (Demitrack and Samuelson, 2016). Additionally, growth factors, such as Wnt, fibroblast growth factor 10 (FGF10) (Lv et al., 2019), and epidermal growth factor (EGF), also play an important role in regulation of self-renewal of gastric stem cells. The Wnt signaling pathway is essential in maintaining the undifferentiated state of progenitor cells in the isthmus region of the gastric corpus, but its activation increases the number of progenitor cells in antrum (Oshima et al., 2006). Antral Lgr5^+^ stem cells are supported and activated by Wnt signaling via at least in part a Frizzled-7 receptor (Flanagan et al., 2017), corpus Mist1^+^ isthmus stem cells can also be activated in part through Wnt 5a pathway (Nienhüser et al., 2020). Wnt pathway activation in gastric stem cells have reported to be associated with gastric carcinogenesis in mice (Oshima et al., 2006; Radulescu et al., 2013).

Though the mesenchymal compartment surrounding the glands is less studied and little understood, immune or stroma cells residing in gastric epithelium also provide an additional environment for gastric stem cells. Cxcl12^+^ endothelial cells and Cxcr4^+^ innate lymphoid cells contributed to the corpus stem cell niche partly through production of Wnt5a from innate lymphoid cells (Hayakawa et al., 2015a). Furthermore, as described above, R-spondin 3 secreted from myofibroblasts is an important component for the antral stem cell niches, and thus predominantly activating Axin2^+^/Lgr5^–^ stem cells (Sigal et al., 2017). Gastrointestinal hormones, such as gastrin and acetylcholine (Ach), may also play unique roles in the antral stem cell niche. Gastrin is secreted from G cells residing near the antral isthmus region, and CCK2R is the receptor of gastrin and its precursor form-progastrin under normal condition. As aforementioned, CCK2R^+^ stem cells have been discovered in this same zone, and progastrin can stimulate the proliferation of CCK2R-expressing stem cells but gastrin dose not (Hayakawa et al., 2015b). Ach generated from tuft cells in gastric epithelium regulates gastric epithelial proliferation and regeneration, as well as the clonal expansion of Lgr5^+^ stem cells via the muscarinic receptor subtype 3 (M3R) (Zhao et al., 2014; Hayakawa et al., 2017b).

Despite the importance of these growth factors and cell components in gastric stem cell niches, the identity of cells providing this niche factors and their interactions remain elusive. Meanwhile, knowledge about the niches surrounding the base of corpus gland is also not clear. Further efforts are needed to characterize the various components involved in the gastric stem cells niche and thus in gastric stem cell biology, in order to elucidate their roles in the regulation of gastric epithelium homeostasis.

## *In vitro* Models of Gastric Stem Cells

Regarding the importance of these niche factors, *in vitro* reconstitution of this specific niche has led to the development of gastric stem cell culture. Some simplified techniques have been developed to build *in vitro* models to investigate stomach stem cells. For instance, Yang et al. built a gastric epithelial cell clone (named KMU-GI2) with sustained growth in a low-calcium medium supplemented with N-acetyl-_L_-cysteine and _L_-ascorbic acid 2-phosphate (Yang et al., 2007). These clones were characterized by high proliferation and differentiation potential, ability of anchorage-independent growth, gap junctional intercellular communication as well as Oct4 expression (one of the key markers of adult stem cells). Recently, Garcia et al. (2017) performed *in vitro* culture of adult gastric mucosal cells within a medium rich in growth factors such as EGF, FGF, and hepatocyte growth factor (HGF). They observed that gastric spherical clones on the surface of gastric fibroblasts grow in a single layer. Those spherical cells harbored molecular markers capable of spontaneously differentiating and were unique to gastric stem cells. Nonetheless, these above models cannot achieve pure and long-lived culture *in vitro* after a number of cells divisions, and whether the identity of stem cells would be changed or not in this culture system is not well determined.

With the deepening understanding of the signals that govern stem cell self-renewal, proliferation and differentiation, tissue-specific organoid models were established successfully, allowing for extensive experimentation with stem cells and their niches (Clevers, 2016). In 2010, Barker et al. (2010b), firstly, found that gastric Lgr5^+^ cell isolated from the gastric glands of Lgr5-EGFP-ires-CreERT2 mice were capable of generating a single-layered epithelial structure, that was then called the gastric organoid. This *in vitro* culture system partially recapitulates the microenvironment of stomach stem cells in the body, and contains all necessary niche factors, including EGF, Noggin, R-spondin, Wnt3A, FGF10, and gastrin (ENRWFG). Specifically, these growth factors supplied in this system act different effects. EGF sustains the continuous self-renewal and proliferation of stem cells and long-term culture (Johnson and Guthrie, 1980; Dembinski and Johnson, 1985). Noggin, an antagonist of BMPs, inhibits differentiation and induces an expansion of bud-domain. R-spondin and Wnt3A are the activators of Wnt pathway that promote growth and inhibits differentiation. FGF10 drives budding event and expansion of multiunit organoids. Gastrin has a mitogenic effect on gastric cells. The same group, further, developed a long-term culture system of human gastric organoids using surgical samples from gastric corpus (Figure 2A; Bartfeld et al., 2015). Cultures from different anatomical regions of the stomach maintain the specific molecular characteristics of their site of origin, and the model conserves stable genetic characteristics and biological behaviors of stem cells during long-term culture. In addition to those growth factors we mentioned above (ENRWFG), nicotinamide (also named vitamin B3) and TGF-β inhibitor (A83-01) are necessary in culture human gastric organoids (ENRWFGNiTi), due to its role in promoting initial organoid formation and extending the life span of organoid, respectively (Bartfeld et al., 2015). Besides, ROCK inhibitor Y-27632 is an optional supplement that can avoid anoikis in the early culture time (Schlaermann et al., 2016). Further, gastric organoids described above can be directed into pit-type organoids with the withdrawal of Wnt activator (Bartfeld et al., 2015). Pit-type gastric organoids undergo changes in morphology (become more cystic with less bud domain) and molecular markers (increased Muc5AC, decreased PGC and MUC6). Applying this culture system, subsequent studies from different research groups have successfully established different gastric organoid models using various marker-labeled gastric stem cells as mentioned above (Troy^+^, Mist1^+^, CCK2R^+^, eR1^+^, Axin2^+^, Bmi1^+^, and AQP5^+^ stem cells) (Table 1). Key points that can be applied to confirm the formation of gastric organoid are summarized in Table 3.

**TABLE 3:** Key points to confirm the formation of gastric organoids.

Index	Description	Method
Morphology	A sealed glandular lumen	Inverted microscope
	Gland-domain buds surrounding a central lumen	Inverted microscope
	Single layer epithelial structure	E-cadherin staining/confocal microscope
Self-renewal capacity	Detection of proliferating cells located at the base of the gland-like domain	EdU staining/fluorescence microscope
	Positive for stem cell markers: Lgr5, CD44, OLFM4	Immunofluorescence/confocal microscope/PCR
Expression of gastric-specific markers	Positive: Organoid form Antrum: Muc5AC (surface mucous cell),TFF1 (surface mucous cell), PAS (surface mucous cell), Muc6 (mucus gland cell), SST (endocrine cell), Gastrin, PDX1 Organoid from Corpus: Muc5AC (surface mucous cell),TFF1 (surface mucous cell), PAS (surface mucous cell), GIF (parietal cell), PGC (chief cell), Muc6 (mucus gland cell), TFF2 (mucous neck cell), SST (endocrine cell) Negative for intestinal markers: Muc2, CDX1/2	PCR, immunofluorescence

*EdU 5-ethynyl-2′-deoxyuridine, Lgr5 leucine-rich repeat containing G protein-coupled receptor, OLFM4 Olfactomedin 4, Muc 5AC mucin 5AC, PAS Periodic acid-Schiff, GIF gastric intrinsic factor, PGC pepsinogen C, Muc 6 mucin 6, TFF1 trefoil factor 1, TFF2 trefoil factor 2, SST somatostatin, PDX1 pancreatic and duodenal homeobox1, Muc2 mucin 2, CDX1/2 caudal-type homeobox 1/2, PCR polymerase chain reaction.*

In contrast to gastric organoid directly derived from stomach tissues, McCracken reported *de novo* generation of organoids from directed differentiation of human pluripotent stem cells (PSCs), through manipulation of stem cell niches targeting the FGF, Wnt, BMP, RA (retinoic acid) and EGF pathways (Figure 2B; McCracken et al., 2014). A detailed protocol for the generation of human antral and fundic gastric organoids from PSCs can refer to Broda TR’s method (Broda et al., 2019). Another research group established a method for generating stomach primordium-like spheroids (named e-ST) from embryonic stem cells (ESCs) (Figure 2C; Noguchi et al., 2015). These spheroids were found to differentiate into mature stomach tissue cells in both the corpus and antrum in a three-dimensional culture system. Gastric organoids generated from PSCs or ESCs are, therefore, a valuable adjunct to *in vitro* studies of human stomach development.

Organoid culture system is a powerful method, but it is important to note its limitation. Current gastric organoids are unable to fully model the *in vivo* environment due to the lack of other cell components (such as mesenchymal cells and immune cells) which also exert an important role in stem cell niches, though coculture organoids with other cells type is a feasible and alternative approach. Considering the high cost of cultivation maintenance, it has become more common to culture organoids in medium with niche factors produced by various cell lines than to use commercial products; thus, experimental variation between scientific groups is inevitable.

## Role of Gastric Stem Cells in Stomach Diseases

**FIGURE 4:**
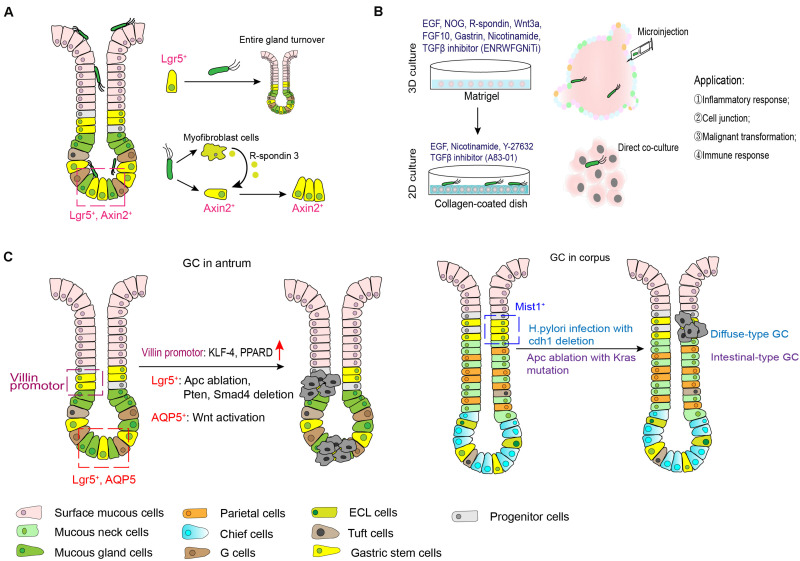
Role of gastric stem cells in *H. pylori* infection and gastric carcinogenesis. **(A)** *H. pylori* can colonize in gastric surface cells, neck regions and even the base of antral glands. Gland-colonized bacteria directly or indirectly expands the stem cell pools. **(B)** Microinjection of *H. pylori* with gastric organoid can mimic the model of *H. pylori* infection *in vitro*, while co-culture model of *H. pylori* and organoid-derived primary gastric epithelial cells are more easier to investigate the pathogenesis of this bacterium. **(C)** The candidate stem cell origin and possible mechanism behind gastric oncogenesis.

The stomach is the most important organ within the gastrointestinal tract, it not only initiates the digestive process but also as a first line of defense against food-borne microbes (Hunt et al., 2015). Stomach diseases, such as gastritis, peptic ulcer, Helicobacter pylori (*H. pylori*) infection and gastric cancer, bothers a lot of people in the world. Gastritis is defined as any histologically confirmed inflammation of the gastric mucosa, and its epidemiology overlaps that of *H. pylori* infection. Longstanding mucosal inflammation leads to the loss of resident gland and the replacement of normal gland by inappropriate glandular unites, which terms as atrophy and metaplasia, respectively. Gastric atrophy and metaplasia are widely recognized as the precursor change for gastric carcinogenesis (Correa et al., 2010; Annibale et al., 2020). With regard to gastric cancer, it is the third leading cause of cancer mortality in the world and remains a major health threat in Asia-Pacific regions (Bray et al., 2018; GBD 2017 Stomach Cancer Collaborators, 2020). However, the mechanism behind gastric carcinogens is still undetermined. Thus, it is clinically important to understand the role of stem cells in stomach diseases. Currently, relevant studies mainly focus on *H. pylori*-associated gastritis and gastric cancer (Figure 4).

### *H. pylori* Infection

Long-term infection of *H. pylori* is one of the key causes of stomach cancer. This might be attributed to persistent mucosal inflammation, epigenetic modification of oncogenes or antioncogenes, and activation of oncogenic signaling pathways following *H. pylori* infection (Conteduca et al., 2013; Amieva and Peek, 2016). In addition to its known damage to differentiated gastric epithelium, available studies have reported that *H. pylori* infection also disturbed homeostasis in the gastric stem cells niche, leading to malignant transformation.

In both mice model and human specimens infected with *H. pylori*, bacteria microcolonies could be visualized in the neck region and base of gastric antral glands. Gland-colonized bacteria directly accelerated Lgr5^+^ gastric stem cell-derived gland turnover at the early stage after infection in the dependence of CagA island (Sigal et al., 2015). The same group further revealed that in addition to directly acting on gastric stem cells, *H. pylori* infection increased expression of gastric myofibroblasts-derived R-spondin 3 expression and expanded the Axin2^+^/Lgr5^–^ cell pool to cause hyperproliferation and gland hyperplasia (Sigal et al., 2017). However, it should be noted that an increase in R-spondin 3 from myofibroblasts was also important to restrict bacteria colonization and promote mucosal regeneration (Sigal et al., 2019). Additionally, apoptotic suppression induced by *H. pylori* CagA via MEK/ERK pathway also contributed to impaired self-renewal of the gastric epithelium (Mimuro et al., 2007). On the other hand, it is well established that long-term infection of *H. pylori* induces a variety of histological changes in gastric mucosa, including oxyntic atrophy, metaplasia and dysplasia. Spasmolytic polypeptide-expressing metaplasia (SPEM) is proposed to encompass TFF2 (trefoil factor family 2)-expressing metaplasia, which is linked to mucosal injury associated with parietal and chief cell lose. Recent study documented that during chronic inflammation induced by this bacterium, some chief cells arising from Lrig1^+^ populations might contribute to SPEM development (Wroblewski et al., 2019). Collectively, the imbalance between gastric epithelial turnover and stem cells differentiation triggers the development of *H. pylori*-associated diseases.

*In vitro* assays with gastric organoid, microinjection of *H. pylori* was used for fundamental studies of *H. pylori* pathogenesis. Upregulation of NF-κB-driven inflammatory response was observed in *H. pylori* infected-organoids (McCracken et al., 2014; Bartfeld et al., 2015; Schumacher et al., 2015), which validated previous results from immortalized gastric cancer cell lines. Meanwhile, in this gastroid system, Wroblewski reported that activated Wnt/β-catenin signaling pathway in a *H. pylori* CagA-dependent manner contributed to mis-localization of tight junction proteins (Claudin-7), leading to disrupted barrier function (Wroblewski et al., 2015). Additionally, application of gastric organoid/immune cell (cytotoxic T lymphocytes and dendritic cells) co-culture system further demonstrated the protective role of programmed death ligand (PD-L1) expression in gastric epithelium in response to *H. pylori*-induced immune injury (Holokai et al., 2019). Instead of 3D culture system, organoid-derived primary gastric epithelial cells cultured in collagen-coated dish (containing EGF, nicotinamide, Y-27632 and A83-01) were more easier to handle *H. pylori* infection without the usage of microinjection (Schlaermann et al., 2016). Therefore, these organoid models greatly simulate the interactions between *H. pylori* and the gastric epithelium or microenvironment, and provide opportunity to uncover mechanisms of malignant transformation induced by this bacterium.

### Gastric Carcinogenesis

Gastric carcinogenesis is a multi-step process, which is correlated to host factors, environmental factors and some specific microbiotas (Tan and Yeoh, 2015; Schulz et al., 2019). Understanding the cells of origin involved in gastric cancer is of clinical significance for guiding development of effective strategies to prevent and treat this disease.

Gastric cancer is classified into intestinal, diffused and mixed types (Lauren classification) based on histological morphology of tumors (Lauren, 1965). Intestinal-type carcinoma undergoes non-atrophic gastritis, atrophic gastritis, intestinal metaplasia, dysplasia, and eventually forms cancer (known as Correa cascade) (Correa, 1992). Metaplasia of the stomach, including spasmolytic polypeptide-expressing metaplasia (SPEM) and intestinal metaplasia (IM), is the tissue injury adaptation and a precursor to the dysplasia (Giroux and Rustgi, 2017). Cell populations contributing to SPEM formation is partially understood. Some evidence indicate a link between SPEM development and mucus neck cells, chief cell transdifferentiation and even the isthmus stem cells in the corpus (Hayakawa et al., 2017a; Mills and Goldenring, 2017; Burclaff et al., 2020). During IM, expression of stem cell marker-Sox2 is reduced while intestinal marker (Cdx1 and Cdx2) emerges ectopically (Tsukamoto et al., 2004); and lineage tracing by following mutations in mitochondria DNA demonstrates the clonal expansion of IM by fission (McDonald et al., 2008). These findings raise the possibility that IM might be the consequence of abnormal differentiation in stem cells that can produce both gastric- and intestinal-type cells. Additionally, the stability and durability of metaplasia also propose that it might be maintained by a self-renewing stem cell. Regarding dysplastic lesions, more recent studies have shown that they were genetically related to metaplastic glands, indicating the clonal origin of dysplasia from metaplasia by field cancerization (Gutierrez-Gonzalez et al., 2011). For diffuse-type gastric cancer, histopathological analyses also have shown that early hereditary diffuse gastric cancer (HDGC) (with *CDH1* mutation) seems to lie within the upper neck of the gastric epithelium (Humar et al., 2007). These available evidence supports the implicit assumption that gastric cancer might arise from gastric stem cells that have potential for multi-directional differentiation potential.

Subsequent studies are identifying some candidate cells from which stomach cancer might originate. As earlier described, Lgr5^+^ gastric stem cells at the base of the antrum in lesser curvature that is a frequent anatomical site for human gastric cancer give rise to all types of epithelial cells. Thus, gastric stem cells in this region might be the potential origin of stomach cancer. Results from a TCGA (The Cancer Genome Atlas) database analysis and immunohistologic staining revealed that intestinal adenocarcinomas of the gastric antrum and gastroesophageal junction were accompanied by the expansion of Lgr5 (Uehara et al., 2013). Ablation of *Apc* (adenomatous polyposis coli) gene in Lgr5-expressing cells leads to macroscopic adenomas and intramucosal well-differentiated carcinoma (Barker et al., 2010b). In line with this, antral stem cells expressing Sox2 or Mist1 may be among the gastric cancer origin cells in the context of *Apc* loss (Sarkar et al., 2016; Sakitani et al., 2017). Subsequent research identified that progression from microadenoma and macroscopic adenoma to invasive intestinal-type gastric cancer in Lgr5^+^ gastric stem cells were accompanied with the deletion of Smad4 and *Pten* in the gastric antrum, while *Smad4* and *Pten* deletions in differentiated cells (including antral pit, parietal and corpus Lgr5^+^ chief cells) failed to initiate tumor growth (Li et al., 2016). However, whether Lgr5 expressing cells in acid-secreting corpus region are cancer cell origin is to be debatable. One study indicated that Lgr5^+^ cells were not the origin for SPEM following the treatment with DMP-777 or L-635 (two chemicals inducing acute loss of parietal cells) (Nam et al., 2012). But, inflammation and inhibition of BMP signaling induced activation of Lgr5^+^ cell in this anatomic region can lead to the development of metaplastic and dysplastic epithelial cell lineages (Ye et al., 2018). Leushacke et al. also found that a subpopulation of chief cells expressing Lgr5 functioned as damage-inducible stems cells effecting gland regeneration following injury and as a source of SPEM under the constitutive activation of Kras (Leushacke et al., 2017). Besides Lgr5^+^ cells, *Klf-4* (Kruppel-like factor 4) disruption or *PPARD* (peroxisome proliferator-activated receptor delta) overexpression in Villin promoter-expressing stem cells spontaneously result in gastric carcinogenesis (Li et al., 2012; Zuo et al., 2019), CCK2R^+^ cells potentially explains the gastrin-mediated effects on gastric cancer progression (Hayakawa et al., 2015b). More recently, AQP5^+^ stem cells are identified as a source of Wnt-driven gastric cancer, and AQP5^+^ tumor cells could reproducibly generate organoids in the absence of exogenous growth factors, indicating the stem potential of this cell populations (Tan et al., 2020). For gastric cancer occurred in the corpus, Mist1^+^ isthmus stem cells can serve as an origin. In combination with Helicobacter species infection, deletion of *Cdh1* gene in Mist1^+^ isthmus stem cells is able to generate diffuse-type gastric cancer; whereas intestinal-type adenocarcinoma can be induced by ablation of *Aps* along with Kras mutation in this group of cells (Hayakawa et al., 2015a). Another two groups of stem cells labeled by *TFF2* mRNA and Lrig1, respectively, do not give rise to metaplasia linage in the corpus (Quante et al., 2010; Choi et al., 2018).

Regarding the development of neuroendocrine tumors in the stomach, enteroendocrine cells, especially the ECL cells, are generally regarded as the cell origin (Waldum and Fossmark, 2018). As stated before, some stem cells contribute to the production of enteroendocrine cells in the gastric epithelium (Table 1), but whether these stem cells can directly give rise to neuroendocrine tumors is not well determined. For example, results from animal models indicated that Lgr5^+^ cells may not be the origin cells for pyloric neuroendocrine carcinomas, though this subtype stem cells give rise to endocrine cells in this region (Vetter et al., 2016). Besides, evidence for the interaction between enteroendocrine cells and stem cells is limited. In small intestine, the enterochromaffin cell that is closely similar to the ECL cell in the stomach has been shown to participate in stem cell dynamics (Sei et al., 2018). For antral stem cells expressed the gastrin receptor CCK2R, they can be stimulated by progastrin but not by amidated gastrin, and this activation resulted in carcinogenesis (Hayakawa et al., 2015b).

On the other hand, cancer initiation is generally thought to commence after mutation in oncogenes or tumor suppressor genes. Renewal of the gastric epithelium is so rapid and frequent that differentiated epithelial cells would not survive for many decades required to achieve the mutational threshold for malignant transformation. In line with this notion, long-lived stem cells appear to the ideal cellular targets for the accumulation of mutations under the action of external environments (such as continuous inflammation and carcinogen stimulation) (Visvader, 2011; Blokzijl et al., 2016). In gastric epithelium, study in mice have documented that Lgr5^+^ epithelial stem cell pool in the antrum was more susceptible to DNA damage than Lgr5^–^ cells (Uehara et al., 2013). Thus, once DNA repair is impaired, increased mutagenesis of genome is possible to initiate carcinogenesis. Another evidence also showed that a deletion mutant of *Apc* in differentiated cells did not give rise to antral tumors, whereas *Apc* deletion in Lgr5^+^ cell did (Barker et al., 2010b). Nevertheless, exactly how gastric cancer is initiated remains unclear, but adult stem cells residing in the gastric epithelium might be the direct source of cancer, given their fundamental properties of multi-potentiality and longevity.

## Implications for Management of Stomach Diseases

Since gastric stem cells in adult tissues can regenerate the resident cell types within a lineage, it might be introduced to harness their potential for regeneration of lost or damaged gastric mucosa. As aforementioned above, gastric stem cells can respond to mucosal injury caused by acute damage and chronic inflammation in animal models (Arnold et al., 2011; Choi et al., 2018; Nienhüser et al., 2020). But currently, there is no data assessing the dynamic changes of stem cells from clinical specimens. Besides, uncovering the effect of gastric mucosal protectants on gastric stem cells is also clinically practical. More importantly, gastric atrophy or intestinal metaplasia that is caused by chronic inflammation is commonly regarded as a point of no return (Annibale et al., 2020), targeted regulation of the differentiation of gastric stem cells might be an effective approach to reversing the damaged mucosa, and even reducing the risk of gastric carcinogenesis. In contrast to benign diseases in the stomach, treatment of patients with malignant poses greater challenges. Aberrant differentiation of gastric stem cells occur during tumorigenesis, development of new drugs (such as small molecule compounds) to inhibit aberrant differentiation might be an effective way to prevent gastric cancer. Additionally, understanding the mechanism behind the transformation from gastric stem cells to cancer stem cells may provide a new insight to solve the problem of tumor recurrence.

## Conclusion and Future Direction

Gastric stem cells play a key role in the dynamic renewal of gastric mucosal epithelium. Linage tracing approach has made breakthroughs that enable understanding of the physiological characteristics of gastric stem cells in their niches, but further efforts are warranted for defining the various components involved in gastric stem cell niches. Establishment of gastric organoid models overcomes the defects associated with multiple-passaged cells lines derived from cancer specimens, thereby laying a foundation for further in-depth investigations into host-bacterial interactions and gastric carcinogenesis. Hyperproliferation of stomach stem cells, induced by *H. pylori* infection, might be one of the mechanisms of oncogenic transformation resulting from this bacterium. Furthermore, disturbance of gastric stem cell pool is involved in the multiple steps of gastric carcinogenesis. In the future, it is clinically significant to uncover the contribution of gastric stem cells to gastric mucosa homeostasis and the development of intestinal metaplasia, dysplasia and final gastric cancer. Further in-depth research is required to reveal the roles of gastric stem cells in stomach diseases and provide new insights for designing strategies to prevent gastric cancer.

## Author Contributions

SX researched data for this manuscript, made a substantial contribution to discussion of content designed the figures, and wrote the manuscript. LZ made a substantial contribution to discussion of content, and reviewed and edited the manuscript. Both authors have read and approved the final manuscript submitted.

## Conflict of Interest

The authors declare that the research was conducted in the absence of any commercial or financial relationships that could be construed as a potential conflict of interest.
